# Contribution of Antigen-Processing Machinery Genetic Polymorphisms to Atopic Dermatitis

**DOI:** 10.3390/life11040333

**Published:** 2021-04-10

**Authors:** Wanda Niepiekło-Miniewska, Łukasz Matusiak, Joanna Narbutt, Alekandra Lesiak, Piotr Kuna, Andrzej Wiśniewski, Piotr Kuśnierczyk

**Affiliations:** 1 Laboratory of Immunogenetics and Tissue Immunology, Hirszfeld Institute of Immunology and Experimental Therapy, Polish Academy of Sciences, ul. Rudolfa Weigla 12, 53-114 Wrocław, Poland; wanda.niepieklo-miniewska@hirszfeld.pl (W.N.-M.); andrzej.wisniewski@hirszfeld.pl (A.W.); 2 Department of Dermatology, Venereology and Allergology, Medical University of Wroclaw, 50-368 Wrocław, Poland; luke71@interia.pl; 3 Department of Dermatology, Pediatric Dermatology and Oncology Clinic, ul. Kniaziewicza 1/5, 91-347 Lódź, Poland; joanna.narbutt@umed.lodz.pl (J.N.); aleksandra.lesiak@umed.lodz.pl (A.L.); 4 2nd Department of Internal Medicine, Medical University of Łódź, al. Kościuszki 4, 90-419 Łódź, Poland; piotr.kuna@umed.lodz.pl; 5 Division of Internal Medicine, Asthma and Allergy, Barlicki University Hospital, Medical University of Łódź, ul. Kopcińskiego 22, 90-153 Łódź, Poland

**Keywords:** atopic dermatitis, genetics, LMP2, LMP7, TAP1, TAP2

## Abstract

Atopic dermatitis (AD) is a chronic and recurrent inflammatory dermatosis. We recently described an association of the *C* allele of the single nucleotide polymorphism (SNP) *rs26618* in the *ERAP1* gene and a synergism of ERAP1 and ERAP2 effects on AD risk. Here, we examined whether polymorphisms of other antigen-presenting machinery genes encoding immunoproteasome components LMP2 and LMP7 and peptide transporter components TAP1 and TAP2 may also affect susceptibility to AD or its outcome. We found that the LMP7 rs2071543*T allele decreased disease risk by about 1.5-fold (odds ratio 0.66, 95% confidence interval 0.44–0.99). On the other hand, the LMP2 rs1351383*C allele reduced the mean age at diagnosis from 23 to 15 years (*p* < 0.001). Similarly, the TAP1 rs1135216*C allele decreased the mean age at diagnosis from almost 20 to 14 years (*p* = 0.033). The results are discussed in light of other reports on the role of these polymorphisms in human disease.

## 1. Introduction

Atopic dermatitis (AD) is a chronic and recurrent inflammatory dermatosis. It is characterized by typical localization and morphology of inflammatory skin lesions and by persistent itching. It mainly affects young children. About a fifth of all mankind suffer from atopic dermatitis, but the prevalence of the disease varies from region to region [1,2,3]. Genetic predisposition was recognized as an important risk factor for the occurrence of allergic diseases at the beginning of the 20th century. Subsequently, more advanced epidemiological studies have provided evidence supporting a genetic predisposition to atopic dermatitis [4]. Epidemiological studies have shown that the risk of atopy in a child is 50% when both parents have atopy, and, when one of the parents has the disease, the risk is approximately 25% [5]. The role of genetic factors in atopic dermatitis is confirmed by observations of twins showing a significantly higher risk of the disease in monozygotic twins, 72–86%, compared to dizygotic twins, 21–23% [1,5,6].

Very few studies on associations of AD with *HLA* class I genes have been published thus far: genome-wide association studies indicating polymorphisms in the HLA class I region [7,8,9], description of direct associations of HLA class I alleles with AD [10,11] and demonstration of HLA-A*02:01-restricted response of CD8+ T cells against autoallergen [12].

HLA class I and class II molecules are the most polymorphic proteins in human body, encoded by 145,884 different alleles (allelefrequencies.net). Their function is to present antigens (in the form of peptides bound to peptide-binding groove) to CD8+ T cells (class I HLA) and CD4+ T cells (class II HLA). Several other proteins serve as antigen-processing machinery in peptide preparation and HLA class I loading [13,14]. Among these, low molecular weight proteins LMP2 and LMP7 are components of immunoproteasome, a multiprotein complex cutting ubiquitinated proteins to peptides; transporter associated with antigen protein subunits TAP1 and TAP2 transfer these peptides from cytosol to endoplasmic reticulum; and endoplasmic reticulum aminopeptidases ERAP1 and ERAP2 trim these peptides making them fit better to peptide binding groove of class I HLA molecule. All these molecules are polymorphic, which influences their activity and substrate specificity [15].

Class I HLA molecules interact not only with CD8+ T cell receptors recognizing presented peptides, but also with killer cell immunoglobulin-like receptors (KIRs) which recognize the presence of HLA on the cell surface [16]. KIRs are also quite polymorphic: they not only have many alleles (6716 according to allelefrequencies.net) but also form many different haplotypes differing in number and kind of *KIR* genes [17].

We described the contributions of several new genes to AD: a protective effect of the *KIR2DS1* gene encoding an activating receptor expressed on the surface of natural killer cells and some T lymphocytes [18]; a protective effect of the HLA-C*05:01 allele [11]; an association of the *C* allele of the single nucleotide polymorphism (SNP) *rs26618* in the *ERAP1* gene T lymphocytes [11]; an effect of the *rs26618* on the enzymatic activity of ERAP1 [11]; and synergism of ERAP1 and ERAP2 effects on AD risk [19]. Here, we checked whether polymorphisms of other antigen-presenting machinery genes, encoding immunoproteasome components LMP2 and LMP7, and peptide transporter components TAP1 and TAP2 may also affect susceptibility to AD or its outcome.

## 2. Materials and Methods

In total, 260 patients diagnosed with AD (mean age 17.89 ± 12.55, range 2–62) and 338 control individuals (mean age 41.31 ± 11.29, range 22–69) were enrolled into our study. Patients were recruited by the Department of Dermatology, Venereology and Allergology, Medical University, Wrocław, by the 1st Department of Dermatology and Venereology, Medical University of Łódź; by the N. Barlicki Medical University Hospital, Łódź; and by the Department of Prevention of Environmental Hazards and Allergology, Medical University of Warsaw. The patients were classified according to the clinical criteria of AD diagnosis proposed by Hanifin and Rajka, which have four major criteria: (i) pruritus; (ii) typical morphology and pattern of eczema; (iii) relapsing course; and (iv) personal or family medical history. Major criteria are complemented by 21 minor criteria. Patients fulfilling at least three major and three minor criteria received an AD diagnosis [20]. The age at diagnosis was chosen rather than the age at disease onset because the latter is not always precisely determined, particularly in chronic disease. The clinical severity of AD was evaluated by the severity scoring of atopic dermatitis SCORAD (Severity Scoring of Atopic Dermatitis) index [21]. SCORAD index data were only available for 203 patients. The maximum achievable score is 103 points. The mean SCORAD was assessed as 37.9 ± 17.9 points (range, 14–93 points). Using the SCORAD index, AD was scored in each patient as mild (<25 points; 31.0% of patients), moderate (25–50 points; 47.3%) or severe (>50 points; 21.7%). The study was specifically approved by the ethics committees of the participating institutions, and written and signed informed consent was obtained from all subjects.

**Figure 1:**
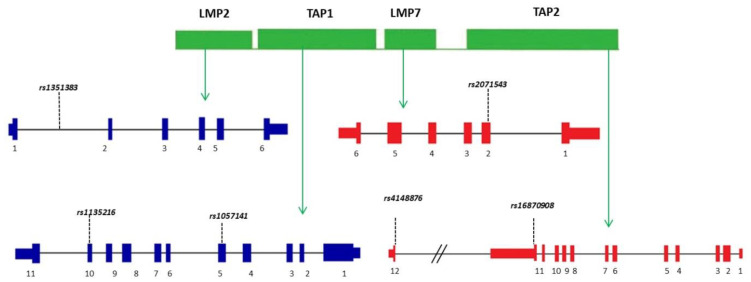
Scheme of *LMP* and *TAP* gene region.

DNA was isolated from venous blood using Invisorb Spin Blood Midi kit (Stratec Molecular, Berlin, Germany) according to the manufacturer’s instructions. Nanodrop 2000 spectrophotometer (Thermo Fisher Scientific, Waltham, MA, USA) was applied to examine the quality and quantity of the extracted DNA samples. All prepared DNA samples had an OD260/OD280 value from 1.7 to 1.9. All SNPs, namely *rs1351383:A>C* (*LMP2*), *rs2071543:G>T* (*LMP7*), *rs1057141:T>C* (*TAP1*), *rs1135216:T>C* (*TAP1*), *rs4148876:G>A* (*TAP2)* and *rs16870908:G>A* (*TAP2*), were selected based on literature data and are shown in Figure 1.

Allelic discrimination for all SNPs was performed using appropriate TaqMan SNP Genotyping Assays (Applied Biosystems, Foster City, USA); the details are shown in Table 1. Amplification reaction was performed in final volume of 10 µL containing 4.5 µL DNA (4 ng/mL), 5 µL TaqMan Universal Master Mix (Applied Biosystems, Foster City, CA, USA) (2×) and 0.5 µL TaqMan Genotyping Assay (Applied Biosystems, Foster City, CA, USA) (20×). PCR conditions were as follows: initial denaturation at 95 °C for 10 min, followed by 40 cycles of denaturing at 95 °C for 15 s and annealing and extension at 60 °C for 1 min. All assays were performed in 96-well plates, including negative template controls. Thermal cycling was carried out on the ViiA7 Real Time PCR system (Thermo Fisher Scientific, Waltham, MA, USA). Data were analyzed with the ViiA7 QuantStudioTM Real Time PCR software version 1.3.

Statistical analysis, including examination of genotype frequencies and correlation of genetic polymorphisms with clinical features, was performed using appropriate tests. Genotype and allele distributions were evaluated and their compatibility with HWE distribution was assessed by means of the Chi-square test. Differences between distributions in particular groups were also evaluated by Chi-square statistic. The odds ratio (OR) and its 95% confidence interval (95% CI) were computed as the measure of effect size. The calculations were performed with software GraphPad Prism 5 (San Diego, CA, USA). Correlation of genetic polymorphisms with clinical features were performed using IBM SPSS Statistics package. Chi-square test and Sidak post hoc tests (F) was used to analyze genetic polymorphisms with age at diagnosis. Chi-square test and Cramer’s V method was used for analysis of genetic variants studied and SCORAD level. Values of statistics for *p* < 0.05 were considered significant.

## 3. Results

Table 2 shows that, among the two *LMP* polymorphisms tested, only *LMP7 rs2071543* was associated with prevalence of AD. The T allele decreased the disease risk by about 1.5 (*p* = 0.039, OR 0.66, CI95% 0.44; 0.99).

However, this SNP did not influence the age at diagnosis, in contrast to *LMP2 rs1351383*, where *C* allele, in both homozygotic and heterozygotic genotypes, was associated with decrease of the mean age at diagnosis from 23 to 15 years (Table 3).

On the other hand, only the heterozygote of *LMP2 rs1351383* was associated with a higher percentage of patients with mild disease and a lower percentage with severe disease, whereas the homozygote CC appeared related to severe disease and AA to both moderate and severe disease (Table 4).

Although TAP1 and TAP2 polymorphisms did not affect susceptibility to AD (Table 2), the *TAP1 rs1135216* heterozygote decreased the mean age at diagnosis to 14 years, whereas such age was higher for rs1135216*T/T (19.64 years, Table 3; C/C homozygote was rare, see Table 2). Again, rs1135216*T/C was associated with less severe disease score (Table 4).

As LMP and TAP genes are encoded in one block in the *LMP2-TAP1-LMP7-TAP2* order in the *HLA* class II region [22], we examined whether the *LMP2-TAP1-LMP7-TAP2* haplotypes marked by SNPs tested here might reveal stronger associations with AD risk, age at diagnosis or severity than each SNP individually. However, no significant associations were found (data not shown).

## 4. Discussion

Proteasome is a multi-catalytic complex present in all cells. Its function is degradation of abnormal or foreign proteins. In cells of the immune system, as well as in other cells upon interferon stimulation, some proteasome subunits are substituted in proteasome by synthesized de novo new subunits LMP2, LMP7 and LMP10 (the latter not tested here). Such a proteasome is called an immunoproteasome (IP) [23]. It should be noted, however, that immunoproteasomes may appear not only in the cells of the immune system and other cells stimulated by interferons during the ongoing immune response, but also in several non-immune tissues under normal conditions [24]. IP subunits have different proteolytic specificities; thus, LMP2 cleaves aspartic or glutamic acid residues, while LMP7 attacks sites after tyrosine and phenylalanine residues [25]. Therefore, effects of polymorphisms in genes encoding these molecules may be different. This may explain why we observed association of *LMP7 (rs2071543*) but not *LMP2 (rs1351383*) SNP with susceptibility to AD, while *LMP2* but not *LMP7* seemed to relate to age at diagnosis and disease severity. Without proper in vitro experiments, we cannot be sure, but we may speculate that LMP7 which cleaves aromatic residues (Tyr and Phe) may produce peptides contributing to induction of AD, whereas LMP2, which cleaves negatively charged residues (Asp and Glu), may affect later stages of disease development. The *LMP2 rs1351383* SNP is located in intron 1 and its biological meaning is not known (https://www.ncbi.nlm.nih.gov/snp/rs1351383#variant_details, 17 March 2021) [26]. However, its *C* allele was shown to give higher mRNA expression than the *A* allele and to bring a higher risk of melanoma, a neoplasm characterized by a higher LMP2 expression [27]. It was also described as predisposing to HCV infection in Han Chinese, however this was published in Chinese language only [28], so the verification of this result on the basis of an abstract in English is not possible. The same authors recently published results seemingly excluding the contribution of *rs1351383* to a risk of cervical intraepithelial neoplasia or cervical cancer which are caused by another virus, HPV [29].

We revealed that the same polymorphism (LMP2 rs1351383*A/A) was associated with later age of diagnosis (and probably later disease onset) but simultaneously with higher disease severity. It is known that factors associated with risk of AD progression and severity include, inter alia, younger age of onset, family history of atopy, filaggrin mutations, urban environment and poly-sensitization and/or allergic multi-morbidity [30]. Therefore, our result seems to state somehow in opposition with the above-mentioned data and require further explanation. It could be speculated here that polymorphism mentioned above might provide some kind of protection from development of more severe course of AD despite of earlier disease onset caused by other factors. In some patients, AD is associated with some comorbidities; most frequent of these are asthma and allergic rhinitis, but vitiligo and some malignancies are also observed [31]. These comorbidities may have different mechanisms than AD, but are nevertheless dependent on adaptive immunity, hence a somewhat similar contribution of *LMP2/7* or *TAP1/2* genes.

Another SNP, in the *LMP7* gene, not examined here, was associated with vitiligo in Indians from Gujarat [32], and a *LMP2* SNP, also not tested by us, was found associated with vitiligo in Saudi Arabia [33]. For *LMP7 rs2071543*, associated here with AD, it was reported that individuals with the *TT* genotype had a 3.5-fold higher ovarian cancer risk in Chinese people and 10-fold higher colon cancer risk in Germans than *TG* and *GG* genotypes [34]. The *T* allele was also found to be associated with cervical cancer similarly to LMP2 rs1351383*A [29], and, in a meta-analysis, also with other gynecological cancers as well as with gastrointestinal cancers [35]. In addition, the LMP7 rs2071543*TT genotype had a negative effect on the treatment of chronic hepatitis C virus-infected patients with pegylated interferon-α and ribavirin [36]. This SNP results in a glutamine to lysine change in position 49. This means a neutral to positive electric charge change which may explain its functional consequences for interactions with proteins and peptides.

A little bit more is known on the *TAP1 rs1135216* associations with diseases. The rs1135216*G (Gly637) allele was described to be strongly associated with vitiligo in a Saudi Arabian population [33], although not in Gujarati Indians [32]. It was also reported to increase the risk of pulmonary tuberculosis in Iranians [37], recurrent respiratory papillomatosis in Mexicans [38] and leprosy in Indians [39]. No association with esophageal squamous cell carcinoma or cervical cancer was found for *rs1135216* or any other *TAP1* SNP tested in Caucasians or Asians in a large meta-analysis encompassing 4719 cases and 4215 controls [40]. Our finding of a lack of association of this SNP with AD was in agreement with results of a recent meta-analysis showing that *rs1135216* was found associated with allergic rhinitis or allergic asthma in some populations, but not with AD [41]. In our AD patients, it affected age at diagnosis and disease severity but not risk. TAP1 rs1135216*T/C genotype was associated with both the earliest diagnosis and the highest proportion of patients with mild disease. rs1135216*T>C results in a change from aspartate (negative charge) to glycine (neutral) in position 637, therefore it may influence protein properties.

The other *TAP1* SNP, *rs1057141*(*T>C*; Ile333Val), had no effect on AD here. A similarly negative result for AD was reported for Asians and Northern Africans by meta-analysis [41]. It was also not associated with pulmonary tuberculosis in Iranians [37]. This is not surprising, as isoleucine and valine have similar biochemical properties.

*TAP2* SNPs were not found associated with AD here. Among *TAP2* SNPs only one, *rs4148876*, was associated with tuberculosis [42] and with several types of cancer, and *TAP2* transcript expression was higher in cancer than in normal tissue, which was also associated with patient survival [40]. *rs16870908* has not been tested thus far in any clinical condition (PubMed search, March 3, 2021) except for our present study.

Our work has several limitations. First, a relatively low numbers of patients and control individuals did not allow for detection of possible associations of *LMP2-TAP1-LMP7-TAP2* haplotypes with AD risk or clinical outcome. Second, as the immunoproteasome produces peptides which may be later presented by HLA class I molecules, the TAP1/TAP2 complex transfers these peptides to the endoplasmic reticulum where they may be bound by HLA-class I molecules, and endoplasmic reticulum aminopeptidases ERAP1 and ERAP2 trim these peptides for better binding by HLA class I molecules, it would be interesting to check whether some combinations of LMP and TAP variants together with optimal ERAP variants provide better peptide repertoire for protection against AD development and/or severity. However, such attempt would require still higher numbers of patients and controls. Third, our limited budget had not permitted us to include class I *HLA* typing to detect effects of antigen-processing machinery polymorphisms in the context of antigen-presenting *HLA* alleles which would also require even higher numbers of tested individuals. Therefore, our results should be confirmed on much larger cohorts of patients and controls.

## 5. Conclusions

In summary, we found interesting associations of polymorphisms of immunoproteasome components LMP2 and LMP7 as well as of the peptide transporter component TAP1 with susceptibility to atopic dermatitis or with its clinical features. Our data are novel, as polymorphisms of *LMPs* and *TAPs* in AD were examined mostly in Asian populations.

## Figures and Tables

**Table 1:** Single nucleotide polymorphisms tested in this work.

Gene	SNP	ID
*LMP2*	*rs1351383* (*A>C*; Intron 1)	C__8848996_10
*LMP7*	*rs2071543* (*G>T*; Gln49Lys)	C__15869253_10
*TAP1*	*rs1057141* (*T>C*; Ile333Val)	C__549926_20
	*rs1135216* (*T>C*; Asp637Gly)	C__531909_20
*TAP2*	*rs4148876* (*G>A*; Arg651Cys)	C__30159972_10
	*rs16870908* (*G>A*; Leu647Phe)	C__34171660_10

**Table 2:** Comparison of *LMP2*, *LMP7*, *TAP1* and *TAP2* single nucleotide polymorphism frequencies in AD and control.

SNP	Genotype/Allele	Patients ;N (%)	Controls ;N (%)	OR	95% CI	χ2	*p*
*LMP2 (A>C)* *rs1351383*	*A/A*	99 (38.1)	123 (36.8)	1			
	*A/C*	122 (46.9)	151 (45.2)	1.01	0.70; 1.43	0.9252	0.629
	*C/C*	39 (15.0)	60 (18.0)	0.81	0.50; 1.31		
	*A*	320 (61.5)	397 (59.4)	1			
	*C*	200 (38.5)	271 (40.6)	0.92	0.72; 1.16	0.5422	0.461
*LMP7 (G>T)* *rs2071543*	*G/G*	207 (84.8)	260 (77.2)	1			
	*G/T*	35 (14.4)	77 (22.5)	**0.58**	**0.37; 0.89**	**7.1014**	**0.028**
	*T/T*	2 (0.8)	1 (0.3)	2.09	0.27; 15.97		
	*G*	449 (92.0)	597 (88.3)	1			
	*T*	39 (8.0)	79 (11.7)	**0.66**	**0.44; 0.99**	**4.2429**	**0.039**
*TAP1 (T>C)* *rs1057141*	*T/T*	181 (69.5)	237 (70.1)	1			
	*T/C*	74 (28.6)	92 (27.2)	1.05	0.73; 1.51	0.4305	0.8064
	*C/C*	5 (1.9)	9 (2.7)	0.76	0.26; 2.16		
	*T*	436 (83.8)	566 (83.7)	1			
	*C*	84 (16.2)	110 (16.3)	0.99	0.73;1.35	0.0030	0.9561
*TAP1 (T>C)* *rs1135216*	*T/T*	183(70.4)	256 (75.7)	1			
	*T/C*	73 (28.1)	78 (23.1)	1.31	0.90; 1.90	2.1675	0.338
	*C/C*	4 (1.5)	4 (1.2)	1.40	0.37; 5.23		
	*T*	439 (84.3)	590 (87.3)	1			
	*C*	81(15.7)	86 (12.7)	1.27	0.91;1.76	1.9925	0.158
*TAP2 (G>A)* *rs4148876*	*G/G*	228 (87.7)	301 (89.1)	1			
	*G/A*	30 (11.5)	35 (10.3)	1.13	0.68; 1.89	0.2893	0.865
	*A/A*	2 (0.8)	2 (0.6)	1.32	0.23; 7.68		
	*G*	486 (93.5)	637 (94.2)	1			
	*A*	34 (6.5)	39 (5.8)	1.14	0.71; 1.84	0.3032	0.582
*TAP2 (G>A)* *rs16870908*	*G/G*	235 (90.4)	302 (89.3)	1			
	*G/A*	23 (8.8)	34 (10.1)	0.87	0.50; 1.52	0.3136	0.855
	*A/A*	2(0.8)	2 (0.6)	1.28	0.22; 7.48		
	*G*	493 (94.8)	638 (94.4)	1			
	*A*	27 (5.2)	38 (5.6)	0.92	0.56; 1.53	0.1052	0.7457

The bold is showing results which are statistically significant.

**Table 3:** Age at diagnosis depending on *LMP* and *TAP* genotype.

SNP	Genotype	Men Age at Diagnosis, Years (SD) *	F		*p*
*LMP2 (A>C)*	*A/A*	23.22 (12.63)		**8.92**	**<0.001**
*rs1351383*	*A/C*	15.04 (12.28)			
	*C/C*	15.05 (9.23)			
*LMP7 (G>T)*	*G/G*	17.52 (12.79)		0.68	0.509
*rs2071543*	*G/T*	20.44 (11.37)			
	*T/T*	15.67 (8.08)			
*TAP1 (T>C)* *rs1057141*	*T/T*	20.62 (13.80)		6.24	0.132
	*T/C*	13.3 (7.88)			
	*C/C*	27.0 (15.56)			
*TAP1 (T>C)* *rs1135216*	*T/T*	19.64 (13.43)		**3.48**	**0.033**
	*T/C*	14.0 (9.46)			
	*C/C*	24.0 (4.24)			
*TAP2 (G>A)* *rs4148876*	*G/G*	18.2 (12.87)		2.82	0.062
	*G/A*	13.36 (9.76)			
	*A/A*	7.33 (3.21)			
*TAP2 (G>A)* *rs16870908*	*G/G*	17.62 (12.52)		0.27	0.973
	*G/A*	18.05 (14.77)			
	*A/A*	14.18 (8.72)			

* Standard deviation (SD). The bold is showing results which are statistically significant.

**Table 4:** Comparison of SCORAD index in patients with different *LMP* and TAP genotypes.

SNP	Genotype	SCORAD			
		Mild (<25)	Moderate (>25 < 50)	Severe (>50)	
*LMP2(A>C)* *rs1351383*	*A/A*	16 (27.6%)	34 (44.7%)	13 (43.3%)	
	*A/C*	37 (63.8%)	32 (42.1%)	10 (33.3%)	***p* = 0.028**
	*C/C*	5 (8.6%)	10 (13.2%)	7 (23.3%)	***V* = 0.18**
*LMP7 (G>T)* *rs2071543*	*G/G*	55 (87.3%)	75 (81.5%)	38 (88.4%)	
	*G/T*	6 (9.5%)	16 (17.4%)	5 (11.6%)	*p* = 0.476
	*T/T*	2 (3.2%)	1 (1.1%)	0 (0.0%)	
*TAP1 (T>C)* *rs1057141*	*T/T*	29 (58.0%)	57 (68.7%)	28 (71.8%)	
	*T/C*	21 (42.0%)	24 (28.9%)	11 (28.2%)	*p* = 0.372
	*C/C*	0 (0.0%)	2 (2.4%)	0 (0.0%)	
*TAP1 (T>C)* *rs1135216*	*T/T*	38 (66.7%)	65 (80.2%)	27 (73.0%)	
	*T/C*	19 (33.3%)	16 (19.8%)	8 (21.6%)	***p* = 0.046**
	*C/C*	0 (0.0%)	0 (0.0%)	2 (5.4%)	***V* = 0.18**
*TAP2 (G>A)* *rs4148876*	*G/G*	53 (80.3%)	83 (85.6%)	38 (91.0%)	
	*G/A*	10 (16.7%)	12 (13.4%)	4 (9.0%)	*p* = 0.508
	*A/A*	2 (3.0%)	1 (1.0%)	0 (0.0%)	
*TAP2 (G>A)* *rs16870908*	*G/G*	55 (87.3%)	85 (88.5%)	40 (90.9%)	
	*G/A*	7 (11.1%)	10 (10.4%)	4 (9.1%)	*p* = 0.487
	*A/A*	1 (1.6%)	1 (1.1%)	0 (0.0%)	

The bold is showing results which are statistically significant.

## Data Availability

Row data are available to interested readers on request.
